# Spiritual Needs of Polish Patients with Chronic Diseases

**DOI:** 10.1007/s10943-014-9863-x

**Published:** 2014-05-01

**Authors:** Arndt Büssing, Iwona Pilchowska, Janusz Surzykiewicz

**Affiliations:** 1 Faculty of Health, Institute of Integrative Medicine, Witten/Herdecke University, Gerhard-Kienle-Weg 4, 58313 Herdecke, Germany; 2 Institute of Cognitive Neuroscience and Social Science, University of Social Sciences and Humanities, Warsaw, Poland; 3 Institute of Social Prevention, Warsaw University, Warsaw, Poland; 4 Catholic University Eichstätt-Ingolstadt, Eichstätt, Germany

**Keywords:** Spiritual needs, Questionnaire, Chronic diseases, Patients, Catholic, Poland

## Abstract

In a cross-sectional survey using standardized questionnaires such as the Spiritual Needs Questionnaire (SpNQ), we analyze unmet spiritual needs of 275 patients with chronic diseases from Catholic Poland. The factorial structure of SpNQ’s Polish version is similar to the primary version and has good internal consistency (Cronbach’s *α* = 0.89). Here, not only *Inner Peace* needs and *Giving/Generativity* needs were of relevance, but also *Religious Needs* and *Existential Needs*. These needs were not significantly associated with life satisfaction, but with interpretations of illness. To address such unmet needs, multi-professional teams should care for patients’ multifaceted needs.

## Introduction

Patients suffering from chronic illness or life-threatening diseases often report unmet needs which are in most cases neither addressed nor even recognized by health care professionals (Büssing and Koenig 2010). In a recent study among US patients with advanced cancer, a majority (72 %) reported that their spiritual needs were supported minimally or not at all by the medical system, and 47 % felt supported minimally or not at all by a religious community (Balboni et al. 2007)—which could be regarded to be in charge for this topic. This is of particular importance, because support by the medical team and pastoral care visits was significantly associated with cancer patients’ quality of life (Balboni et al. 2007, 2010). Moreover, advanced cancer patients who “received less spiritual care than desired” had significantly more depressive symptoms and less meaning and peace (Pearce et al. 2011). Thus, also the care for patients’ spiritual needs is an important aspect of an adequate health care. Yet, it seems unclear who might be in charge for this specific care. In secular societies where several individuals have turned away from institutional religiosity, the chaplain might not be the first contact person for a-religious patients. In German tumor patients, a majority wanted their medical doctor to be interested in their spiritual orientation (Frick et al. 2006). A survey among German patients with chronic pain conditions revealed that 23 % talked with a chaplain/priest about their spiritual/religious needs and 20 % had no partner to talk with, while for 37 %, it was important to talk with their medical doctor about these needs (Büssing et al. 2009b). Thus, health care professionals might be faced with situations they are not trained for.

What are the concrete spiritual needs patients may ask for? A recent conceptual framework for research and clinical practice categorized four (interconnected) core dimensions of psychosocial and spiritual needs (Büssing and Koenig 2010), i.e., connection, peace, meaning/purpose, and transcendence, which can be attributed to the underlying categories of social, emotional, existential, and religious. Using the Spiritual Needs Questionnaire (SpNQ), in predominantly secular German patients with chronic diseases, particularly secular spiritual needs such as *Need for Inner Peace* and *Giving/Generativity* were of outstanding importance, particularly for cancer patients, while *Existential Needs* (*Reflection/Meaning*) or *Religious Needs* were of lower importance (Büssing et al. 2010, 2012). Using the same instrument, also in predominantly atheistic patients from Shanghai, *Giving/Generativity* and *Inner Peace Needs* scored highest, while *Religious Needs* and the *Reflection/Release Needs* scored lower (Büssing et al. 2013a, b). But what about societies with vital religiosity? Will they have a similar pattern of spiritual needs? To address this, we intended to investigate patients with chronic diseases from Poland.

Poland is situated in the area of cultural and religious borderland, in the sphere of Latin and Greek-Slavonic influence but with different influences from communist ideology and in the last year lasting stronger impact of secularization processes. Currently one can note an exchange of values in the Christian Churches and civil society. Polish society is faced with different degrees of sacral tension, spirituality and mysticism, different dogmatic attitudes, different theologies and ecclesiologies, and also ideologies. Originally, Polish religiosity displayed an extremely “Church oriented” structure. The low level of religious knowledge has never prevented people from having a strong identification with the Roman Catholic Church. “To be Polish means to be Roman Catholic,” the famous stereotype formed during the partition of Poland in the eighteenth century remained lively until recently. Religious affiliation was a very important indicator of national identity (Grabowska 2001; Mariański 2000). Studies have shown that up to 97 % of the population of 38 million inhabitants identify themselves as Roman Catholics (Boguszewski 2012; CBOS 2009; Zarzycka 2008).

Here, we report on specific spiritual/religious needs of the Polish patients with chronic diseases. We again used the SpNQ, which is used in its Polish version, and analyzed correlations with specific measure of religiosity, life satisfaction, and interpretation of illness. Our main interest was to analyze which variables were associated with *Religious Needs*, because in a previous study, we found that in German patients with chronic diseases *Religious Needs* were positively associated with spiritual well-being and life satisfaction, while *Existential Needs* and *Inner Peace* needs were correlated with a lack of spiritual well-being and life satisfaction (Büssing et al. 2013a, b).

## Methods

### Participants

All individuals were informed of the purpose of the study, were assured of confidentiality, and gave informed consent to participate. The patients were recruited consecutively by a psychologist and educators in Oncology Hospital in Wieliszew and in Department of Social Welfare in the province of Warsaw. Demographic information of these patients is presented in Table 1.

**Table 1:** Characteristics of 275 patients

Variables	Mean (%)
Gender (%)	
Women	74
Men	26
Age [years (mean, SD)]	56 ± 16
Family status (%)	
Married	54
Divorced	26
Widowed	20
Educational level (%)	
Basic	12
Professional	20
Medium	42
Higher	25
Denomination (%)	
Christian	100
Spiritual/religious self-categorization (%)	
R+S+	78
R+S−	7
R−S+	2
R−S−	13
Underlying diseases (%)	
Cancer	35
Chronic pain diseases	10
Diabetes mellitus	16
Other chronic conditions (including asthma bronchiale and multiple sclerosis)	40
Life Satisfaction scores	
Life Satisfaction (mean, 0–100)	65 ± 13
Escape from Illness (mean, 0–100)	57 ± 25
Religiosity scores	
SpREUK Search (mean, 0–100)	66 ± 24
SpREUK Trust (mean, 0–100)	69 ± 21
SpREUK Reflection (mean, 0–100)	68 ± 27
Positive emotions toward God (mean, 0–100)	70 ± 24
Negative emotions toward God (mean, 0–100)	28 ± 23
SQS Religious Attitudes (mean, 7–35)	27 ± 7
SQS Ethical Sensitivity (mean, 13–58)	29 ± 4
SQS Harmony (mean, 11–44)	22 ± 5

Individuals provided informed consent to participate by returning a completed questionnaire which did not ask for names, initials, addresses, or clinical details (with the exception of a diagnosis). The internal review boards in the persons of the Directorate Institutions and psychologists working in these institutions approved the survey. The study did not provide financial incentives to patients. All completed the questionnaires by themselves.

### Measures

All items of the respective instruments were translated by a bi-language scientist and critically discussed with a committee of Polish psychologists, theologists and medical doctors, and the primary author of the SpNQ. Because cultural equivalence is not guaranteed, the team decided to avoid the back-translation procedure. Instead, to ensure linguistic equivalence, unclear phrases were discussed and adjusted (with respect to cultural specifics and with reference to the intended construct) with the input of the developing author to achieve the best fitting translation suited for the Polish context.

#### Spiritual Needs Questionnaire

To measure patients’ psychosocial and spiritual needs, we used the SpNQ in its Polish version. In its primary version, the instrument differentiates four main factors (Büssing et al. 2010, 2012):

1. *Religious Needs* (Cronbach’s *α* = 0.92), i.e., praying for and with others, and by themselves, participate at a religious ceremony, reading of spiritual/religious books, turn to a higher presence;
2. *Existential Needs* (*Reflection/Meaning*) (*α* = 0.82), i.e., reflect previous life, talk with someone about meaning in life/suffering, dissolve open aspects in life, talk about the possibility of a life after death, etc.;
3. *Need for Inner Peace* (*α* = 0.82), i.e., wish to dwell at places of quietness and peace, plunge into the beauty of nature, finding inner peace, talking with other about fears and worries, devotion by others;
4. *Need for Active Giving/Generativity* (*α* = 0.74) which addresses the active and autonomous intention to solace someone, to pass own life experiences to others, and to be assured that your life was meaningful and of value.

All items were scored with respect to the self-ascribed importance on a 4-point scale from disagreement to agreement (0, not at all; 1, somewhat; 2, very; 3, extremely). The higher the scores, the stronger the respective needs are.

#### SpREUK-15

The contextual SpREUK-15 questionnaire measures SpR attitudes and convictions of patients dealing with chronic diseases (Büssing et al. 2005, 2010). It differentiates three factors (Büssing 2010):

1. *Search* scale, or search (for support/access to SpR), deals with patients’ intention to find or have access to a spiritual or religious resource, which may be beneficial for coping with illness, and with their interest in spiritual or religious issues (insight and renewed interest).
2. *Trust* scale, or trust (in higher guidance/source), is a measure of intrinsic religiosity; the factor deals with patients’ conviction that they want to be connected with a higher source, and with their desire to be sheltered and guided by that source, whatever may happen to them.
3. *Reflection* scale, or reflection (positive interpretation of disease), deals with a patient’s cognitive reappraisal of his or her life because of illness and subsequent attempts to change (i.e., reflecting on what is essential in life, to change aspects of life or behavior, looking for opportunities for development, and believing that the illness has meaning).

The SpREUK-15 scores items on a 5-point scale from disagreement to agreement [0, does not apply at all; 1, does not truly apply; 2, do not know (neither yes nor no); 3, applies quite a bit; 4, applies very much]. The scores were referred to a 100 % level (transformed scale score). Scores >50 % indicate higher agreement (positive attitude), while scores <50 % indicate disagreement (negative attitude).

#### Self-description Questionnaire of Spirituality

The *Self*-*description Questionnaire of Spirituality* (SQS) is an instrument tested first in Polish individuals (Heszen-Niejodek et al. 2003) and was used as an external measure sensitive for spiritual activities of Polish individuals. The scale uses originally 20 items and differentiates three factors, i.e.,

1. *Religious Attitudes* (i.e., “faith allows me to survive difficult periods in my life” and “while making decisions, I rely on my religious beliefs”),
2. *Ethical Sensitivity* (i.e., “react when someone is being hurt” and “care about other people’s situations”), and
3. *Harmony* (i.e., “I am part of the world” and “while thinking about my life I experience peace and happiness”).

However, when testing this scale in our sample, explorative factor analysis indicated four main factors and four items which loaded weakly on the respective factors (<0.5). These items were thus eliminated. The resulting 17-item version of the instrument (SQS-17) with its two main scales *Religious Attitudes* and *Ethical Sensitivity*, and the third scale *Peace/Harmony* with two sub-constructs, has a very good reliability coefficient (Cronbach’s *α* = 0.90) and explains 68 % of variance. For this analysis, we used the SQS-17 version. The SQS-17 scores on a 5-point Likert scale ranging from “not at all” to “very much.” The sum of the subscales indicates overall spirituality.

#### Positive Emotions (Associated with God)

To measure positive or negative emotions associated with God, we used a 12-item scale which was not yet validated for the Polish population. The instrument addresses positive emotions with 6 items (i.e., Happiness/Joy, Love, Affection, Security, Shelter, Confidence/Trust), negative emotions with 5 items (i.e., Guilt, Punishment, Failure, Fear, Anger/Rage), while 1 item addresses a person’s disinterest in God. Within this sample, the sub-scale measuring positive emotions has a very good internal reliability (alpha = .95), and the sub-scale measuring negative perceptions a good internal reliability (alpha = .85). These items were scored on a 5-point scale from disagreement to agreement [0, does not apply at all; 1, does not truly apply; 2, do not know (neither yes nor no); 3, applies quite a bit; 4, applies very much]. The score was referred to a 100 % level (transformed scale score).

#### Life Satisfaction

Life satisfaction was measured using the *Brief Multidimensional Life Satisfaction Scale* (BMLSS) (Büssing and Fischer 2009) which refers to Huebner’s “Brief Multidimensional Students” “Life Satisfaction Scale” (Huebner et al. 2004; Zullig et al. 2005). The items of the BMLSS address intrinsic (Myself, Life in general), social (Friendships, Family life), external (Work situation, Where I live), and prospective dimensions (Financial situation, Future prospects). The internal consistency of the instrument was good (Cronbach’s *α* = 0.87) (Büssing et al. 2009a, b). Here, we included two further items addressing patients’ health situation and abilities to deal with daily life concerns. Each item was introduced by the phrase “I would describe my level of satisfaction as …” and scored on a 7-point scale from dissatisfaction to satisfaction [0, terrible; 1, unhappy; 2, mostly dissatisfied; 3, mixed (about equally satisfied and dissatisfied); 4, mostly satisfied; 5, pleased; 6, delighted]. The BMLSS-10 sum score refers to a 100 % level (“delighted”). Scores >50 % indicate higher life satisfaction, while scores <50 % indicate dissatisfaction.

#### Escape from Illness

The 3-item scale *Escape from Illness* is an indicator of an escape-avoidance strategy to deal with illness (i.e., “fear what illness will bring,” “would like to run away from illness,” “when I wake up, I don’t know how to face the day”) (Büssing et al. 2006). The items were scored on a 5-point scale from disagreement to agreement [0, does not apply at all; 1, does not truly apply; 2, do not know (neither yes nor no); 3, applies quite a bit; 4, applies very much]. Scores >50 % indicate an escape attitude.

#### Interpretation of Illness

The interpretation of illness was measured with eight items according to Lipowski’s “Meaning of Illness” (Lipowski 1970), a scale which was recently validated (Büssing and Fischer 2009). This interpretation of illness scale (IIS) includes positive interpretations (i.e., challenge, value) and strategy-associated interpretations (i.e., relieving break of life, Call for help), but also guilt-associated interpretations (i.e., Punishment, Weakness/failure) and fatalistic negative interpretations (i.e., Threat/Enemy, Interruption of life). The items were scored on a 5-point scale from disagreement to agreement [0, does not apply at all; 1, does not truly apply; 2, do not know (neither yes nor no); 3, applies quite a bit; 4, applies very much].

### Statistical Analyses

The research team performed reliability (Cronbach’s coefficient *α*) and exploratory factor analyses (principal component analysis using Oblimin rotation with Kaiser’s normalization), analyses of variance, correlation, and regression analyses with SPSS 20.0. Confirmatory factor analysis was performed using AMOS 21.

The team judged *p* < .05 as significant. With respect to the correlation analyses, we regarded *r* > 0.5 as a strong correlation, an *r* between 0.3 and 0.5 as a moderate correlation, an *r* between 0.2 and 0.3 as a weak correlation, and *r* < 0.2 as no or a negligible correlation.

## Results

### Participants

As shown in Table 1, patients’ mean age was 56 ± 16; 74 % were women and 26 % men. Most were married and had a medium educational level. All patients had chronic diseases, predominantly cancer (35 %), diabetes mellitus (16 %), chronic pain diseases (10 %), and other chronic conditions.

Polish patients were 100 % Catholics; 78 % regarded themselves as religious and spiritual (R+S+), 7 % as religious but not spiritual (R+S−), 2 % as not religious but spiritual (R−S+), and 13 % as neither religious nor spiritual (R−S−). In line with this, all religiosity indices (SpREUK, SQS, and positive emotions toward God) were in the upper range (Table 1).

Life satisfaction mean sores were expressed in a moderate range (65 ± 13), indicating that they are mostly satisfied, while Escape from Illness scores were in the intermediate range (57 ± 25) (Table 1).

### Reliability and Exploratory Factor Analyses

None of the items from the primary SpNQ item pool had to be removed because of a weak corrected item-total correlation; however, two items were deleted during the processor of factorial analyses (item N4W and N6W). As shown in Table 2, the 18-item Polish SpNQ had a good internal consistency (Cronbach’s *α* = 0.89). Factor analysis of the questionnaire revealed a Kaiser–Mayer–Olkin value of 0.87, which indicates as a measure for the degree of common variance that the item pool is suitable for a factorial validation. Explorative factor analysis indicated that, as stated above, two items had to be removed, i.e., item N6W (“plunge into the beauty of nature”), which would load best on the *Giving/Generativity* factor, and N4W (“reflect previous life”) because of a low factor loading. The resulting five factors would counted for 68 % of variance, with internal consistency coefficients (Cronbach’s *α*) ranging from 0.59 to 0.91. The respective factors are in line with the factors of the original version of the SpNQ, i.e., *Religious Needs*, *Existential Needs*, *Peace Needs*, and *Giving/Generativity*. However, the existential items of the Polish version split into two sub-constructs of *Existential Needs*, i.e., meaning (*α* = 0.80) and relief (*α* = 0.59). Particularly, this *Relief* sub-construct, which would lack item N16W (“forgive someone from a distinct period of life”), was unsatisfactory, and thus we tested a 4-factor solution.

**Table 2:** Factorial structure of the Polish SpNQ

	Mean	SD	Difficulty index (1.58/3 = 0.53)	Corrected item–scale correlation	Alpha if item deleted (*α* = 0.895)	Factors					Original SpNQ factor
						I	II	III	IV	V	
*Religious Needs (Eigenvalue* *=* *6.6; α* *=* *0.91)*											
N21W participate at a religious ceremony	1.88	1.28	0.63	0.625	0.886	0.920					RN
N20W pray for yourself	1.90	1.25	0.63	0.653	0.885	0.919					RN
N23W turn to a higher presence (i.e., God)	1.92	1.25	0.64	0.635	0.886	0.849					RN
N19W someone prays for you	1.66	1.27	0.55	0.700	0.883	0.787					RN
N22W read religious/spiritual books	1.17	1.22	0.39	0.583	0.888	0.748					RN
N18W praying with someone	1.02	1.21	0.34	0.629	0.886	0.615					RN
*Existential Needs: meaning (Eigenvalue* *=* *2.0; α* *=* *0.79)*											
N11W talk with someone about the question of meaning in life	1.23	1.23	0.41	0.618	0.886		0.761				EN
N12W talk with someone about the possibility of life after death	0.94	1.20	0.31	0.518	0.890		0.754				EN
N5W dissolve open aspects of life	1.22	1.23	0.41	0.357	0.895		0.722				EN
N10W find meaning in illness and/or suffering	1.46	1.24	0.49	0.525	0.889		0.605				EN
N2W talk about fears and worries	1.61	1.21	0.54	0.456	0.892		0.560				IP/EN
N16W forgive someone from a distinct period of life	1.39	1.25	0.46	0.495	0.890		0.540				EN
N4W reflect previous life	1.48	1.11	–	–	–	–	–	–	–	–	EN
*Giving/Generativity (Eigenvalue* *=* *1.4; α* *=* *0.78)*											
N26W pass own life experiences to others	1.66	1.24	0.55	0.397	0.894			0.885			GG
N27W be assured that life was meaningful and of value	2.05	1.12	0.68	0.490	0.891			0.757			GG
N15W solace someone	1.73	1.11	0.58	0.587	0.888			0.645			GG
N13W turn to someone in a loving attitude	1.58	1.11	0.53	0.578	0.888			0.549			GG
*Peace Needs (Eigenvalue* *=* *1.2; α* *=* *0.71)*											
N7W dwell at a place of quietness and peace	1.97	1.11	0.66	0.298	0.896				0.868		IP
N8W find inner peace	2.10	1.07	0.70	0.465	0.891				0.770		IP
N6W plunge into beauty of nature	1.46	1.17	–	–	–	–	–	–	–	–	IP
*Marker items*											
N17W be forgiven	1.47	1.30	–	–	–	–	–	–	–	–	–
N25W feel connected with family	2.38	0.96	–	–	–	–	–	–	–	–	–

Principal component analysis; Oblimin rotation with Kaiser normalization; the respective five factors would explain 68 % of variance; factor loadings <0.30 are not depicted

*SpNQ* Spiritual Needs Questionnaire, *EN* Existential Needs, *IP* Inner Peace, *RN* Religious Needs, *GG* Giving/Generativity

The resulting 4-factors are in line with the original version of the SpNQ and would explain 62 % of variance with internal consistency coefficients ranging from 0.71 to 0.91 (Table 2). The item difficulty of the 18 items [1.58 (mean value)/3] of the items was 0.53; all values were in the acceptable range from 0.2 to 0.8.

**Fig. 1:**
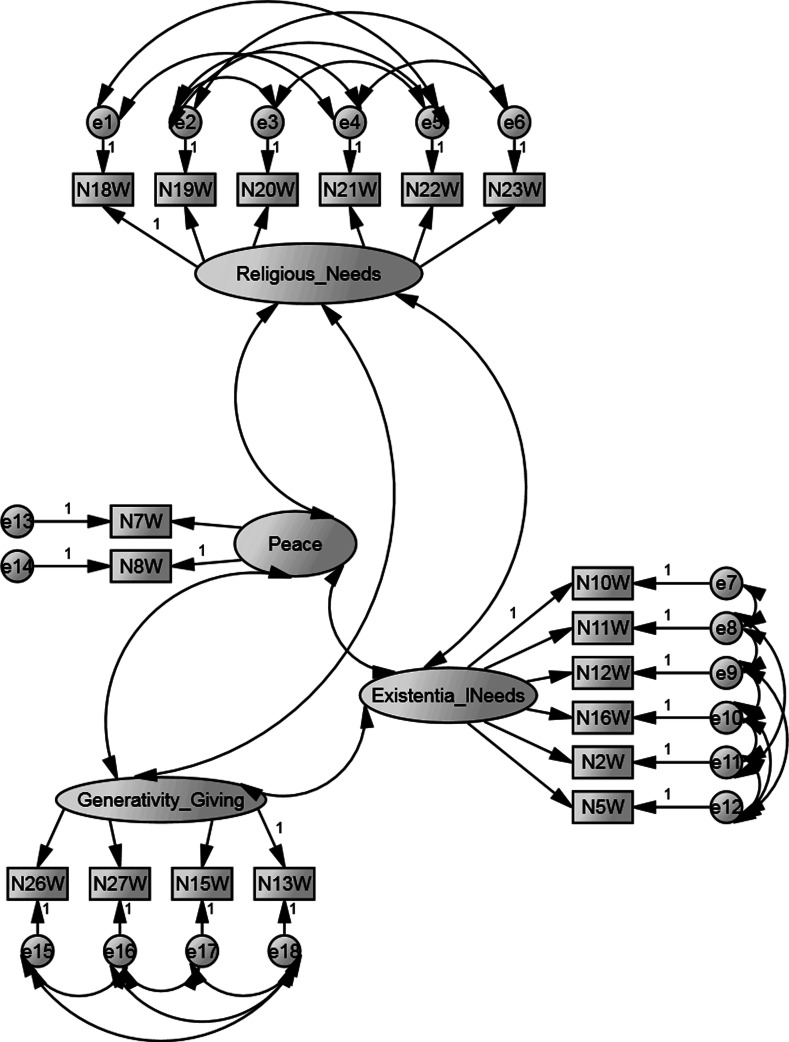
Graphic representation of the tested model

Using confirmatory factor analysis, we examined the internal structure of the Polish version of the SpNQ. Received model correctly matched to the data (χ2 (108) = 147.76, *p* < .01, CMIN/DF = 1.37, CFI = 0.88, RMSEA = 0.038). The model allows us to explain about 93.4 % of the variance of the analyzed variables (Fig. 1). Detailed analysis of the items showed that all items correlated significantly (*p* < .001) and strongly with the intended factors. When interpreting the factor loadings (Table 3), it was observed that the 2-item *Peace* factor was characterized only by item N8W and weakly by item N7W. The factor *Giving/Generativity* was explained best by items N13W, N15W, N27W, and weakly by N26W. The *Existential Needs* factor was defined as the average by items N2W, N10W, N11W, N16W, and weakly by items N5W and N12W. *Religious Needs* were explained as the average by items N19W, N20W, N21W, N23W, and weakly by items N18W and N22W.

**Table 3:** Value of factorial goods and items correlations with the respective factors

	Peace	Giving/Generativity	Existential Needs	Religious Needs	Item to factor correlation*
N7W	**0.029**	0.001	0.002	0.000	0.547
N8W	**0.935**	0.026	0.075	0.003	0.980
N13W	0.003	**0.276**	0.054	0.005	0.781
N15W	0.003	**0.249**	0.048	0.005	0.791
N26W	0.000	**0.035**	0.007	0.001	0.531
N27W	0.002	**0.218**	0.042	0.004	0.648
N2W	0.004	0.025	**0.132**	0.003	0.623
N10W	0.003	0.020	**0.107**	0.003	0.636
N11W	0.003	0.020	**0.105**	0.003	0.686
N12W	0.002	0.013	**0.067**	0.002	0.577
N5W	0.001	0.003	**0.017**	0.000	0.406
N16W	0.004	0.025	**0.130**	0.003	0.601
N18W	0.000	0.002	0.004	**0.026**	0.682
N19W	0.001	0.020	0.030	**0.219**	0.901
N20W	0.001	0.011	0.015	**0.115**	0.890
N21W	0.001	0.015	0.022	**0.164**	0.884
N22W	0.000	0.005	0.007	**0.050**	0.695
N23W	0.001	0.010	0.015	**0.108**	0.850

Significant differences were highlighted in bold

* *p* < .001

### Correlation Analysis

Correlation analyses (Table 4) revealed moderate interconnections between the respective needs factors, at least *Giving/Generativity* was strongly associated with *Existential Needs*.

**Table 4:** Correlation analyses

	Religious Needs	Existential Needs	Giving/Generativity	Peace
Religious Needs	1	0.460**	0.460**	0.303**
Existential Needs		1	0.523**	0.325**
Giving/Generativity			1	0.355**
Peace Needs				1
SpREUK Search	0.700**	0.256**	0.285**	0.202**
SpREUK Trust	0.643**	0.265**	0.312**	0.121
SpREUK Reflection	0.668**	0.150	0.254**	0.118
SQS Religious Attitudes	0.711**	0.203**	0.283**	0.134
SQS Ethical Sensitivity	0.414**	0.267**	0.446**	0.191**
SQS Harmony	0.302**	0.020	0.181**	−0.016
Positive emotions toward God	0.578**	0.159**	0.231**	0.163**
Negative emotions toward God	−0.249**	−0.106	−0.016	0.113
Life satisfaction	0.016	−0.107	0.135	−0.050
Escape from Illness	−0.010	0.200**	0.014	0.099
Illness: Value	0.333**	0.139	0.302**	0.146
Illness: Challenge	0.080	−0.108	0.037	0.155
Illness: Threat/Enemy	0.011	0.282**	0.190**	0.160**
Illness: Interruption	−0.066	0.210**	0.199**	0.046
Illness: Punishment	−0.109	0.049	−0.041	−0.058
Illness: Weakness	−0.069	0.138	0.058	−0.029
Illness: Relieving break	0.011	0.008	0.001	0.104
Illness: Call for help	0.192**	0.208**	0.142	0.215**

** *p* < .01 (Pearson)

*Religious Needs* were strongly associated with SpREUK’s Search, Trust and Reflection sub-constructs, with SQS’s Religious Attitudes, and with positive emotions toward God; moreover, there were moderate correlations also with SQS’s Ethical Sensitivity and Harmony, and the illness interpretation Value (Table 4).

*Existential Needs* were weakly associated with SpREUK’s Search and Trust, with SQS’s Ethical Sensitivity, with Escape from Illness, and disease interpretations such as Threat/Enemy and Call for help.

*Giving/Generativity* was moderately associated with Ethical Sensitivity, religious Trust, and the illness interpretation Value.

*Peace Needs* were weakly associated with spiritual Search, and the interpretation of illness as a Call for help.

None of the respective needs was significantly (*p* < .01) associated with patients’ life satisfaction; however, Escape from Illness was weakly associated only with *Existential Needs.*

### Expression of Psychosocial and Spiritual Needs Among Polish Patients

To analyze which needs were of particular relevance, we measure the intensity of respective needs among the patients. It was striking that all needs were expressed relatively high, particularly *Peace Needs* and *Giving/Generativity* (Table 5).

**Table 5:** Expression of spiritual needs in patients with chronic diseases

	Religious needs	Existential needs	Giving/generativity	Peace
All				
Mean	1.59	1.31	1.75	2.03
SD	1.03	0.85	0.89	0.97
*Gender*				
Women				
Mean	1.76	1.40	1.82	2.09
SD	0.98	0.85	0.87	0.96
Men				
Mean	1.14	1.06	1.57	1.85
SD	1.06	0.80	0.92	1.01
*F* value	**20.1**	**8.7**	**4.1**	3.3
*p* value	**<.0001**	**.003**	**.043**	.071
*Age categories*				
≤40 years				
Mean	1.25	1.31	1.39	1.81
SD	1.02	0.97	0.94	1.21
41–50 years				
Mean	1.41	1.38	1.67	1.88
SD	1.02	0.80	0.96	0.94
51–60 years				
Mean	1.72	1.33	1.79	2.05
SD	1.03	0.87	0.86	0.94
61–70 years				
Mean	1.46	1.13	1.71	2.11
SD	1.01	0.87	0.85	0.89
>70 years				
Mean	2.00	1.43	2.09	2.17
SD	0.95	0.75	0.77	0.92
*F* value	**4.4**	1.1	**4.0**	1.2
*p* value	**.002**	n.s.	**.003**	n.s.
*Family status*				
Alone				
Mean	1.73	1.35	1.70	2.02
SD	1.08	0.89	0.91	1.02
With partner				
Mean	1.48	1.28	1.80	2.02
SD	0.98	0.82	0.86	0.94
*F* value	**4.1**	0.6	0.9	0.0
*p* value	**.044**	n.s.	n.s.	n.s.
*Disease*				
Cancer				
Mean	1.52	1.37	1.69	1.94
SD	1.06	0.90	0.90	0.99
Chronic pain				
Mean	2.04	1.67	1.95	2.20
SD	1.02	0.90	0.89	0.84
Diabetes				
Mean	1.53	1.19	1.71	1.77
SD	0.91	0.75	0.92	1.07
Other chronic				
Mean	1.58	1.21	1.77	2.16
SD	1.05	0.82	0.86	0.94
*F* value	1.9	2.6	0.07	2.2
*p* value	n.s.	.054	n.s.	.085

Significant differences were highlighted in bold

With respect to specific socio-demographic data, women had the highest scores compared to men, particularly with respect to *Religious Needs* (*F* = 20.1; *p* < .001). Age had an influence on the variance of *Religious Needs* and *Giving/Generativity*. Interestingly, those living alone had higher *Religious Needs* (*F* = 4.1; *p* = .044), while all other needs did not significantly differ. Patients with chronic pain had in trend higher *Existential Needs* and *Peace Needs* when compared to patients with other chronic diseases (Table 5).

### Predictors of Spiritual Needs

Because we empirically investigated several variables that could have influenced patients’ spiritual needs, we performed stepwise regression analyses to identify the most significant predictors (Table 6). The variables which were recognized to have a significant impact on the respective needs included gender, age, SpREUK’s search, trust and reflection scales, the self-described Spirituality (SQS) scales, positive emotions toward God, interpretations of illness, and Escape from Illness.

**Table 6:** Regression analyses with spiritual needs as dependent variables (stepwise method)

	Beta	*T*	*p*	Collinearity statistics^a^	
				Tolerance	VIF
*Dependent variable: Religious Needs (R* ^2^ *=* *0.573)*					
Model 4					
(Constant)		−6.138	.000		
SQS: Religious Attitudes	0.299	3.851	.000	0.274	3.648
SpREUK: Search	0.270	3.739	.000	0.316	3.164
Positive Interpretation of Illness	0.252	3.429	.001	0.304	3.285
Illness: Threat/Enemy	0.093	2.205	.028	0.929	1.077
*Dependent variable: Existential Needs: meaning (R* ^*2*^ *=* *0.232)*					
Model 5					
(Constant)		−0.513	.608		
Illness: Threat/Enemy	0.379	6.879	.000	0.937	1.067
SpREUK: Trust	0.231	3.656	.000	0.713	1.402
Illness: Challenge	−0.195	−3.532	.000	0.933	1.072
Male gender	−0.133	−2.370	.019	0.904	1.106
SQS: Ethical Sensitivity	0.142	2.315	.021	0.756	1.323
*Dependent variable: Giving/Generativity (R* ^*2*^ *=* *0.361)*					
Model 6					
(Constant)		−5.392	.000		
SQS: Ethical Sensitivity	0.336	6.373	.000	0.888	1.126
Illness: Threat/Enemy	0.158	2.455	.015	0.593	1.687
Illness: Value	0.296	5.352	.000	0.805	1.242
Age	0.245	4.846	.000	0.964	1.038
Illness: Interruption	0.179	2.724	.007	0.572	1.749
Illness: Relieving break	−0.112	−2.154	.032	0.906	1.104
*Dependent variable: Peace Needs (R* ^*2*^ *=* *0.114)*					
Model 4					
(Constant)		1.687	.093		
Illness: Call for help	0.209	3.126	.002	0.752	1.330
SQS: Ethical Sensitivity	0.152	2.548	.011	0.945	1.058
Illness: Punishment	−0.170	−2.625	.009	0.801	1.249
Illness: Threat/Enemy	0.151	2.356	.019	0.822	1.216

^a^Because the regression coefficients may be compromised by collinearity, we checked the variance inflation factor (VIF) as an indicator for collinearity. VIF > 10 is indicative of high collinearity

As shown in Table 5, *Religious Needs* can be predicted best (*R* ^2^ = 0.57) by Religious Attitudes (SQS), spiritual Search, and Reflection (Positive Interpretation of Illness), with a weak positive influence, however, also of interpretation of disease as a Threat/Enemy.

*Existential Needs* were predicted best (*R* ^2^ = 0.23) by disease interpretation Threat/Enemy, religious Trust, and negatively by illness interpretation Challenge; male gender had an additional (negative) influence, while Ethical Sensitivity had an additional positive influence.

*Giving/Generativity* needs were predicted best (*R* ^2^ = 0.36) by Ethical Sensitivity (SQS), disease interpretation Value, and by patients’ age, with a further positive influence of negative disease interpretations such as Threat/Enemy and Interruption, and a weak negative modulating influence of disease as a Relieving break.

*Peace* needs were predicted with weak predictive power (*R* ^2^ = 0.11) best by disease interpretation Call for help and negatively by disease interpretation Punishment, and further variables.

Since the regression coefficients may be compromised by collinearity, we checked the variance inflation factor (VIF) as an indicator for collinearity. A VIF higher than 10 is indicative of high collinearity. Results suggest that a VIF was not present in the respective models.

## Discussion

The SpNQ was developed to measure a wide range of psychosocial and spiritual needs and was intended to be used also in secular societies and atheistic individuals. Thus, one may expect that the interpretation whether a specific need has a religious connotation or not may depend on the cultural context. Similarly, specific needs could be related to the development of inner peace states, or to an existential search for meaning—which may also relate to states of inner peace.

In contrast to the more secular German patients with chronic diseases (36 % without any religious denomination) (Büssing et al. 2010, 2012) or chronic patients from Shanghai (77 % had no religious denomination) (Büssing et al. 2013b) which were all investigated with the same instrument, all Polish patients enrolled in this study were Christians. To them, not only *Inner Peace* needs and *Giving/Generativity* were of relevance, but also *Religious Needs* and *Existential Needs* which were of lower relevance in the more secular patients from Germany or Shanghai.

The primary structure of the SpNQ remained stable in the Polish version. However, the split of the *Existential Needs* factor into two sub-constructs (i.e., Meaning and Relief) was unsatisfactory, while a 4-factor solution was convincing and fits best with the structure of the original instrument. Moreover, one item of the *Peace* needs scale had to be removed because of a weak factor loading, and another item (N2W “talking with others about fears and worries”), which was primarily related to the peace category, refers to the existential category. Thus, the Polish *Peace Needs* are represented by two items only. Relying on the method of confirmatory factor analysis, we were able to approve the structural model of the SpNQ. We were also able to identify items which address topics which may relate also to other categories (i.e., N5W “dissolve open aspects of life”), but are nevertheless part of the respective factor. The fact that in the Polish sample, some items may load better on other factors or should be eliminated due to weak factor loading may depend on their specific religious and cultural background, or also to the fact that in this sample a relatively large proportion of patients with diabetes mellitus were included, a disease which is not primarily burdening or fatal.

How are these factors related to variables of spiritual well-being and life satisfaction? In German patients with chronic diseases, *Religious Needs* were positively associated with spiritual well-being and life satisfaction, while *Existential Needs* and *Inner Peace* needs were correlated with a lack of spiritual well-being and life satisfaction (Büssing et al. Büssing et al. 2013a). In contrast, in Polish patients with chronic diseases, *Religious Needs* were not a matter of high or low life satisfaction. It seems that these specific needs point to a vital resource they can rely on. In fact, regression analyses revealed that the best predictors of Polish patients’ *Religious Needs* were their Religious Attitudes (i.e., “faith allows me to survive difficult periods in my life” and “while making decisions, I rely on my religious beliefs”), also their search to find access to a spiritual/religious resource which might be beneficial to cope with illness, and finally reflection in terms of a positive interpretation of illness which implies the ability to see illness as something of value, as a hint to change attitudes and behavior.

*Existential Needs* were of lowest relevance in Polish patients; however, patients with chronic pain diseases had in trend higher *Existential Needs* than patients with cancer of other chronic diseases. It might be that either the provision of health care or their satisfaction with the treatment effects is less satisfactory to them, and they could enunciate more specific what they need than other patients. Indeed, the strongest predictor was the interpretation of illness as a Threat/Enemy, while the ability to see illness also as a Challenge was a negative predictor. Even if they are in need for additional support, they seem to have religious trust, which was a strong positive predictor, too.

In contrast, *Giving/Generativity* was predicted best by Ethical Sensitivity, which means concrete empathic reactions toward others; this is obviously in line with the conceptual background of the *Giving/Generativity* scale (i.e., patients’ intention to solace someone, to pass own life experiences to others, but also to be assured that life was meaningful and of value). The main focus of these specific needs is to care for others. This attitude may refer to Erikson’s (1974) psychosocial development stage “generativity” which is the ability to care for others and guide the next generation—and to approve that one’s own life has been of value to others, and thus meaningful. Interestingly, disease was regarded as something of value (to develop), which may indicate processes of “spiritual transformation.”

The two items which would make up the *Peace* factor of the Polish version (i.e., dwell at places of quietness and peace, and find inner peace) are weakly associated only with SpREUK’s Search and illness as a Call for help. This search for a helpful resource to cope (and also Call for help) obviously intends to generate peaceful situations, which are not a matter of reduced life satisfaction or Escape from Illness. Best predictors of these *Peace Needs*, although with weak predictive power, were illness as a Call for help and negative interpretations such as Punishment and Threat/Enemy, and Ethical Sensitivity. From a theoretical point of view, it makes sense that negative disease perceptions are associated with patients needs to find peace and rest.

A limitation of this study was the cross-sectional design which precludes causal interpretations; longitudinal studies are needed to substantiate the findings of this study. Moreover, one may argue that the symptoms of the respective chronic diseases and their impact on life expectancy or daily life activity may differ. While we agree that this is true, it is nevertheless of importance to assess whether a specific need is of particular relevance for patients with specific diseases (i.e., cancer, chronic pain) or a more general need which may occur also in patients with less fatal or burdening diseases (i.e., diabetes mellitus). Although we were unable to state significant differences, *Existential Needs* were in trend higher in patients with chronic pain diseases and lower in patients with diabetes mellitus. Further studies should enroll larger samples of patients with specific diseases. In this study, the relatively large group of “other chronic conditions” was too heterogeneous.

## Conclusion

The Polish version of the SpNQ is similar to the primary version, has a good internal and external validity, and can be used for further research in a predominantly Catholic population. Also in Polish patients with chronic diseases, *Peace* needs and *Giving/Generativity* had the highest relevance, while *Religious Needs* were of strongest relevance, too, and *Existential Needs* of lower relevance. Thus, secular spiritual needs are of strongest relevance in patients both with and without a specific religious denomination. To address these needs, multi-professional teams (i.e., chaplains, nurses, medical doctors, psychologists, social workers) should care for the multifaceted needs of their patients/clients, particularly in secular societies where chaplains might not be the primary contact of patients, but also in societies which started to change with trends of secularization such as Poland.
